# Assessing the impact of 25-hydroxyvitamin concentrations on mortality in chronic diarrhea: a cross-sectional analysis

**DOI:** 10.3389/fmed.2025.1508439

**Published:** 2025-02-14

**Authors:** Pengyu Li, Menglong Zou, Ziming Peng

**Affiliations:** ^1^School of Hunan University of Traditional Chinese Medicine, Changsha, Hunan, China; ^2^The First Hospital of Hunan University of Traditional Chinese Medicine, Changsha, Hunan, China; ^3^Fangchenggang Hospital of Traditional Chinese Medicine, Fangchenggang, Guangxi, China

**Keywords:** chronic diarrhea, serum 25-hydroxyvitamin D, all-cause mortality, NHANES, L-shaped association

## Abstract

**Background:**

The aim of this study was to assess the relationship between serum 25-hydroxyvitamin levels and all-cause mortality in patients with chronic diarrhea.

**Methods:**

We carried out a cross-sectional study using information drawn from the National Health and Nutrition Examination Survey (NHANES). To assess mortality outcomes, we compared our data with records from the National Death Index as of December 31, 2011. The NHANES data were used to determine mortality outcome. We used a Cox regression model-based approach to analyze the relationship between serum 25-hydroxyvitamin concentrations and mortality in chronic diarrhea patients.

**Results:**

A total of 2,972 participants with chronic diarrhea were included in our study, 488 cases of all-cause mortality were recorded. The study showed an L-shaped relationship between 25-hydroxyvitamin concentrations and all-cause mortality with a threshold of 73.40 nmol/L. On the left side of the threshold, each 1-unit increase in 25-hydroxyvitamin concentrations was associated with a 2.2% reduction in the risk of all-cause mortality (HR 0.978; 95% CI: 0.969, 0.987); however, on the right side of the threshold, there was no significant correlation between 25(OH)D concentrations and all-cause mortality.

**Conclusion:**

Serum 25-hydroxyvitamin D levels showed an L-shaped association with all-cause mortality in patients with chronic diarrhea, with 73.40 nmol/L as the potential threshold. However, because this was a cross-sectional study, only an association, not a causal relationship, can be inferred. Further prospective studies are needed to confirm these findings and explore the potential impact of vitamin D supplementation on mortality outcomes.

## 1 Introduction

Chronic diarrhoea is a common and complex digestive disorder usually defined as an abnormal bowel condition that lasts more than 4 weeks (1, 2), it manifests as an increase in loose or watery stools. Although patients may describe diarrhea as an increase in the frequency of bowel movements or an urgency to pass stools, a more accurate definition should be based on the abnormal shape of the stools rather than the frequency of bowel movements (1, 3, 4). Chronic diarrhea, with its complex and varied causes, including dysfunction such as inflammatory bowel disease, irritable bowel syndrome, malabsorption syndromes, infections, and medication side-effects, it is estimated that approximately 17% of the world’s population is affected by this disease, and it greatly impacts their quality of life (5, 6). In addition, chronic diarrhea can lead to dehydration, electrolyte imbalance, weight loss and a host of other serious health issues.

Vitamin D, a fat-soluble vitamin, primarily exists in the body as 25-hydroxyvitamin D [25(OH)D], playing a crucial role in calcium and phosphorus metabolism regulation (7). The primary source of vitamin D is synthesized in the skin through sunlight exposure (8). Nevertheless, with shifts in lifestyle and a growing trend toward sun avoidance, vitamin D deficiency is on the rise worldwide, making it one of the most widespread nutritional shortfalls today (9). 25(OH)D is recognized for its vital role in strengthening the immune system, protecting against infections and autoimmune diseases (10). It also inhibits inflammatory responses and enhances intestinal barrier function (11, 12). In addition, it regulates the microbiota in the gut to relieve diarrhea symptoms (13). When 25(OH)D levels are insufficient, the intestinal barrier function may weaken, increasing inflammation. This can lead to excessive water and electrolyte loss, triggering or worsening diarrheal symptoms (14). Studies show that conditions linked to chronic diarrhea, like inflammatory bowel disease (IBD), are often associated with vitamin D deficiency (15). Supplementing with vitamin D may contribute to symptom relief and play a beneficial part in the management of diarrhea. A comprehensive review indicates that elevated levels of 25(OH)D is notably linked to a reduced risk of mortality among individuals with colorectal cancer (16). Research suggests that individuals with vitamin D deficiency and liver disease may have a heightened risk of developing cancer (17, 18). However, the relationship between vitamin D deficiency and all-cause mortality in patients with chronic diarrhea has not been fully explored, and most of the available literature focuses on specific diseases, such as IBD, with fewer studies directly analyzing chronic diarrhea as a widespread disease group. Considering the potential role of vitamin D in immune system regulation, intestinal barrier function, and diarrhea symptom relief, the aim of this study was to investigate the correlation between serum 25(OH)D levels and all-cause mortality in patients with chronic diarrhea and to assess its possible protective role in this specific group.

## 2 Methods

### 2.1 Data sources

Our study used a cross-sectional design to analyze data from the National Health and Nutrition Examination Survey (NHANES) conducted by the Centers for Disease Control and Prevention (CDC) between 2005 and 2010. The NHANES was designed using a complex multistage stratified probability sampling methodology designed to provide a representative sample covering individuals residing outside of institutions in the United States. The NHANES was designed to provide a representative sample of individuals living outside of U.S. institutions. All participants were assessed at a Mobile Examination Center (MEC). Our study includes participants aged 20 and above who completed a questionnaire on gut health. Bowel activity is evaluated with the Bristol Stool Form Scale (BSFS), which includes a color-coded chart displaying pictures and detailed descriptions of seven different stool types, from hard, lumpy stools (type 1) to completely liquid stools (type 7).

To assess participants’ bowel habits, they were instructed to examine this card and indicate the number that best reflects their typical or most frequent stool type. To determine participants’ bowel habits, they were instructed to examine this card and indicate the number that best reflects their typical or most frequent stool type. The BSFS primarily identifies types 6 (loose, mushy stools) and 7 (entirely liquid stools) as indicative of diarrhea. As well as this, the frequency of bowel movements was assessed with the question, “How many bowel movements do you usually have each week?” Individuals whose predominant stool type is categorized as type 6 or 7 are defined as having chronic diarrhea. A non-chronic diarrhea participant, an individual with inflammatory bowel disease, celiac disease, or colon cancer, and a participant with missing 25(OH)D results and missing covariates were excluded from the study.

During the study, all participants signed informed consent forms, and the publicly available NHANES data did not contain any identifying information about the patients. A secondary data cohort study was not required to have additional institutional ethics board approval because it was a prospective cohort study.

### 2.2 Determination of mortality

We tracked participants’ all-cause mortality status until December 31, 2011, using the 2005–2010 NHANES Linked Mortality File. Mortality data for each participant was retrieved through a connection to the National Death Index (NDI).

### 2.3 Measurement of serum 25(OH)D concentrations

The DiaSorin RIA kit (Stillwater MN) was used to measure 25(OH)D concentrations in the NHANES 2005–2006. For the 2007–2010 cycle, the CDC and Prevention used liquid chromatography–tandem mass spectrometry (LC–MS/MS) to determine 25(OH)D concentrations in order to ensure accuracy and consistency. By using regression, NHANES measured 25(OH)D concentrations using radioimmunoassay (RIA) during the 2005–2006 period and converted them into measurements similar to those obtained using LC–MS/MS (19). Following are the clinical practice guidelines of the Endocrine Society’s (20):< 50.00 nmol/L indicates deficiency, 50.00–74.99 nmol/L indicates insufficiency, and ≥ 75.00 nmol/L indicates sufficiency.

### 2.4 Assessment of covariates

This study analyzed factors such as age, gender, race, physical activity, household income, education levels, smoking, diet, body mass index (BMI), and health conditions. In order to define stroke, angina, congestive heart failure, heart attack, cancer, and coronary heart disease, participants answered the following question: “Has your doctor or another healthcare professional ever diagnosed you with congestive heart failure, coronary heart disease, angina, or a heart attack?” Diabetes was diagnosed by self-report from a doctor, hemoglobin A1c (HbA1c) ≥6.5%, or the use of diabetes medications or insulin. An assessment of hypertension was based on self-report, systolic blood pressure of 130 mmHg, diastolic blood pressure of 80 mmHg, or antihypertensive medications taken. Serum cotinine levels indicated smoke exposure: <3.00 ng/mL was considered non-smoking, and > 10 ng/mL was considered smoking. Physical activity was defined based on the individual’s typical weekly physical activities, including vigorous activities that significantly increase heart rate or breathing, lasting at least 10 min.

### 2.5 Statistical analysis

Taking into account the Clinical Practice Guidelines of the Endocrine Society, we divided 25(OH)D concentrations into three groups: <50.00 nmol/L, 50.00–74.99 nmol/L, and ≥ 75.00 nmol/L. Mean and standard deviation (SD) were used to report continuous data, and percentages were used to report categorical data. We used weighted linear regression and weighted chi-square tests to analyze differences in quantitative and qualitative variables between the three groups. A multivariate Cox regression model was used to calculate the hazard ratio (HR) and 95% confidence intervals (CI). The models were constructed as follows: model 1 (unadjusted); model 2 (adjusted for race, gender, and age); and model 3 (adjusted further for race, gender, age, educational attainment, poverty-to-income ratio, BMI, stroke, angina pectoris, coronary heart disease, myocardial infarction, cancer, congestive heart failure, diabetes mellitus, alcohol consumption, hypertension, physical activity, cotinine levels, dietary fiber, carbohydrate, fat, protein, sugar and total energy intake). In addition, subgroup analyses were performed to investigate the association between 25(OH)D and all-cause mortality, and interaction *p*-values were examined in each subgroup. Following the reviewers’ suggestions, we reclassified age into the following three groups: ≥20 years and < 45 years, ≥45 years and < 60 years, and ≥ 60 years. This age grouping adjustment helps to more accurately assess the effect of 25(OH)D on all-cause mortality in different age groups. Furthermore, the specific relationship between 25(OH)D and all-cause mortality was further explored by weighted generalized additivity modeling and smoothed curve-fitting methods. An analysis of the data was carried out utilizing Empower Stats software (Version 4.0) and R software (Version 4.1.6), with *p*-values <0.05 deemed statistically meaningful.

## 3 Results

### 3.1 Baseline characteristics

**Figure 1:**
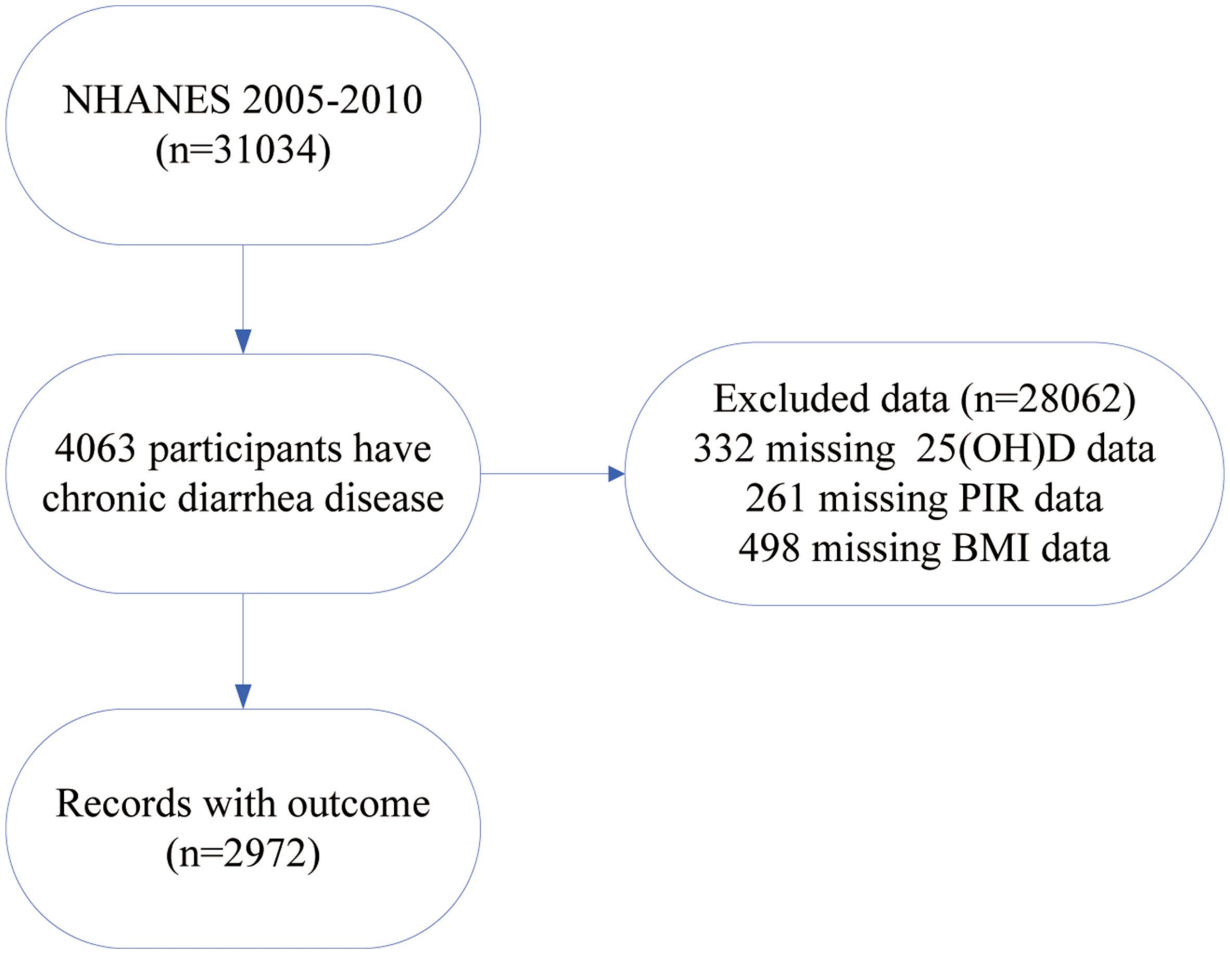
Flowchart for research participant selection.

Out of the 31,034 participants from NHANES 2005–2010, we selected 4,063 individuals with chronic diarrhea for our analysis. Exclusions were as follows: (1) participants missing 25(OH)D data (*n* = 332), (2) participants missing poverty-income ratio (PIR) data (*n* = 261), and (3) participants missing BMI data (*n* = 498). As a result, we included 2,972 participants in the study (Figure 1). We divided the included individuals into three groups and analyzed their socio-demographic characteristics along with specific disease conditions (Table 1).

**Table 1:** Baseline characteristics of chronic diarrhea participants in NHANES data from 2005 to 2010.

	Serum 25(OH)D concentrations (nmol/L)			*P*-value
	<50.00	50.00–74.99	≥75.00	
*N* (%)	949	1,189	834	
Age (years), Mean (SD)	48.21 (17.07)	50.44 (17.49)	52.14 (19.18)	<0.01
Gender, N (%)				<0.01
Male	427 (44.99%)	625 (52.57%)	347 (41.61%)	
Female	522 (55.01%)	564 (47.43%)	487 (58.39%)	
Race, N (%)				<0.01
Mexican American	195 (20.55%)	245 (20.61%)	70 (8.39%)	
Other Hispanic	72 (7.59%)	78 (6.56%)	35 (4.20%)	
Non-Hispanic White people	293 (30.87%)	714 (60.05%)	676 (81.06%)	
Non-Hispanic Black people	348 (36.67%)	117 (9.84%)	33 (3.96%)	
Other Race - Including Multi-Racial	41 (4.32%)	35 (2.94%)	20 (2.40%)	
Education level, N (%)				<0.01
Less than high school	321 (33.83%)	323 (27.17%)	146 (17.51%)	
High school	229 (24.13%)	278 (23.38%)	203 (24.34%)	
More than high school	399 (42.04%)	588 (49.45%)	485 (58.15%)	
PIR, N (%)				
≤1.3	329 (34.67%)	323 (27.17%)	183 (21.94%)	
>1.3, ≤ 3	325 (34.25%)	382 (32.13%)	242 (29.02%)	
>3	295 (31.09%)	484 (40.71%)	409 (49.04%)	
BMI, kg/m^2^, Mean (SD)	30.90 (7.39)	29.17 (6.20)	27.12 (5.23)	<0.01
Cotinine level, Mean (SD)	75.00 (141.27)	55.25 (132.19)	49.98 (120.10)	<0.01
Congestive heart failure, N (%)				0.01
Yes	38 (4.00%)	34 (2.86%)	14 (1.68%)	
No	911 (96.00%)	1,155 (97.14%)	820 (98.32%)	
Coronary heart disease, N (%)				0.39
Yes	50 (5.27%)	60 (5.05%)	33 (3.96%)	
No	899 (94.73%)	1,129 (94.95%)	801 (96.04%)	
Angina, N (%)				0.79
Yes	27 (2.85%)	39 (3.28%)	28 (3.36%)	
No	922 (97.15%)	1,150 (96.72%)	806 (96.64%)	
Heart attack, N (%)				0.59
Yes	47 (4.95%)	55 (4.63%)	33 (3.96%)	
No	902 (95.05%)	1,134 (95.37%)	801 (96.04%)	
Stroke, N (%)				0.33
Yes	44 (4.64%)	41 (3.45%)	30 (3.60%)	
No	905 (95.36%)	1,148 (96.55%)	804 (96.40%)	
Alcoholic ≥ 4 drinks/day (%)				<0.01
Yes	610 (64.28%)	843 (70.90%)	661 (79.26%)	
No	339 (35.72%)	346 (29.10%)	173 (20.74%)	
Stroke, N (%)				0.33
Yes	44 (4.64%)	41 (3.45%)	30 (3.60%)	
No	905 (95.36%)	1,148 (96.55%)	804 (96.40%)	
Hypertension, N (%)				0.27
Yes	528 (55.64%)	660 (55.51%)	436 (52.28%)	
No	421 (44.36%)	529 (44.49%)	398 (47.72%)	
Cancer, N (%)				<0.01
Yes	82 (8.64%)	116 (9.76%)	120 (14.39%)	
No	867 (91.36%)	1,073 (90.24%)	714 (85.61%)	
Diabetes, N (%)				<0.01
Yes	168 (17.70%)	140 (11.77%)	72 (8.63%)	
No	781 (82.30%)	1,049 (88.23%)	762 (91.37%)	
Physical activity, N (%)				0.09
Yes	205 (21.60%)	299 (25.15%)	212 (25.42%)	
No	744 (78.40%)	890 (74.85%)	622 (74.58%)	
Daily dietary intake, Mean (SD)				
Fiber (g)	14.78 (9.10)	17.20 (10.54)	17.28 (9.57)	<0.01
Carbohydrate (g)	253.52 (123.31)	264.05 (118.34)	257.91 (114.74)	0.12
Total fat (g)	77.56 (44.69)	81.81 (45.75)	78.58 (40.04)	0.06
Protein (g)	78.54 (41.10)	84.91 (42.35)	80.47 (36.65)	<0.01
sugar (g)	114.63 (77.23)	119.30 (75.50)	118.12 (70.29)	0.34
Energy (kcal)	2079.61 (967.21)	2178.15 (952.42)	2106.99 (856.25)	0.04

Among the 2,972 chronic diarrhea patients, the average age was 50.26 years, and 52.93% were female. Of these, 55.01% had 25(OH)D deficiency and 47.44% had 25(OH)D insufficiency. Participants with higher 25(OH)D levels tended to be older, predominantly non-Hispanic white people, had a higher proportion of females, consumed more dietary fiber, and were more likely to be educated with higher incomes. They were less likely to have congestive heart failure, cancer, or diabetes but were more likely to be overweight (BMI ≥ 25) (*p* < 0.05). However, differences in myocardial infarction, hypertension, stroke, coronary heart disease, and angina were not statistically significant.

### 3.2 Relationships of 25(OH)D concentrations with mortality

All-cause deaths were recorded in the included survey in a total of 488 cases. To investigate whether 25(OH)D status has an independent effect on mortality, we performed three Cox regression models (Table 2). 25(OH)D concentrations were not significantly associated with mortality in the unadjusted model. However, according to the adjusted model, 25(OH)D concentrations were negatively associated with all-cause mortality. Model 3 reported HR and 95% CI of 1.00 (reference group), 0.65 (0.50, 0.85), and 0.56 (0.39, 0.80) for 25(OH)D levels ranging from severe deficiency to adequacy, with a trend *p*-value <0.01, respectively.

**Table 2:** HRs (95% CI) for mortality according to serum 25(OH)D concentrations among participants.

	Serum 25(OH)D concentrations (nmol/L)			*P* for trend
	<50.00	50.00–74.99	≥75.00	
Model 1	1.00 (Reference)	0.68 (0.52,0.89)	0.68 (0.44,1.05)	0.82
Model 2	1.00 (Reference)	0.53 (0.41,0.68)	0.46 (0.31,0.68)	<0.01
Model 3	1.00 (Reference)	0.65 (0.50,0.85)	0.56 (0.39,0.80)	<0.01

Model 1: Non-adjusted model. Model 2: Adjusted for age, gender, race. Model 3: Adjusted for gender, age, race, educational attainment, family income as a continuous variable, BMI as a continuous variable, congestive heart failure, coronary heart disease, angina, heart attack, stroke, cancer, diabetes, alcohol intake, hypertension, physical activity, cotinine level, dietary fiber, dietary carbohydrates, dietary fats, dietary protein, dietary sugars, and dietary energy.

### 3.3 Detection of linear relationships

**Figure 2:**
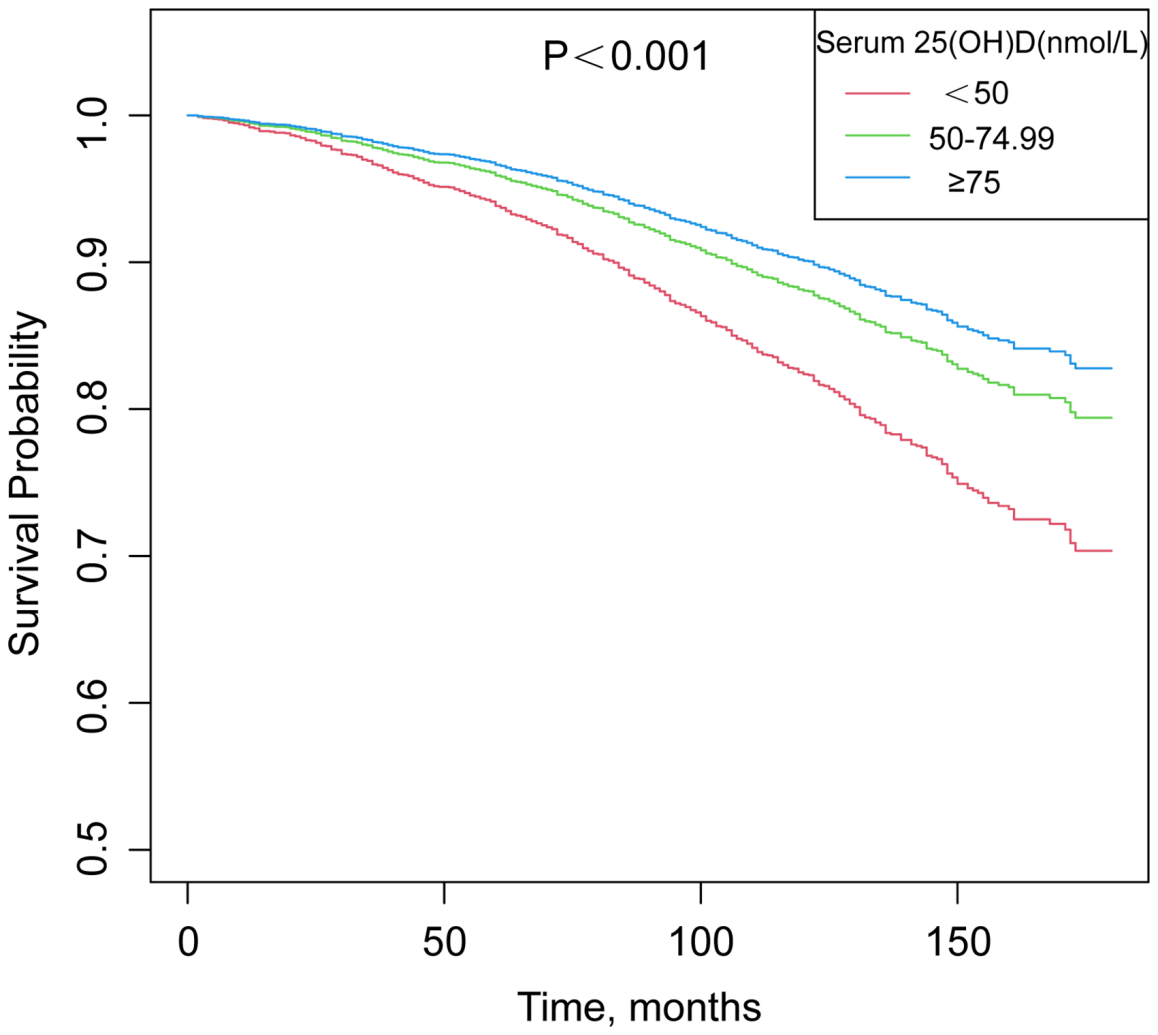
Survival curves for the association between serum 25(OH)D concentrations and chronic diarrhea.

According to Figure 2, overall survival rates increase as 25(OH)D increases. A high concentration group has the highest survival rate, while a low concentration group has the lowest. However, the survival curves within each group are not entirely smooth. The rate of decline for each curve is not completely consistent, which may reflect the influence of other underlying variables. This implies that a non-linear relationship may exist between survival rate and time.

**Figure 3:**
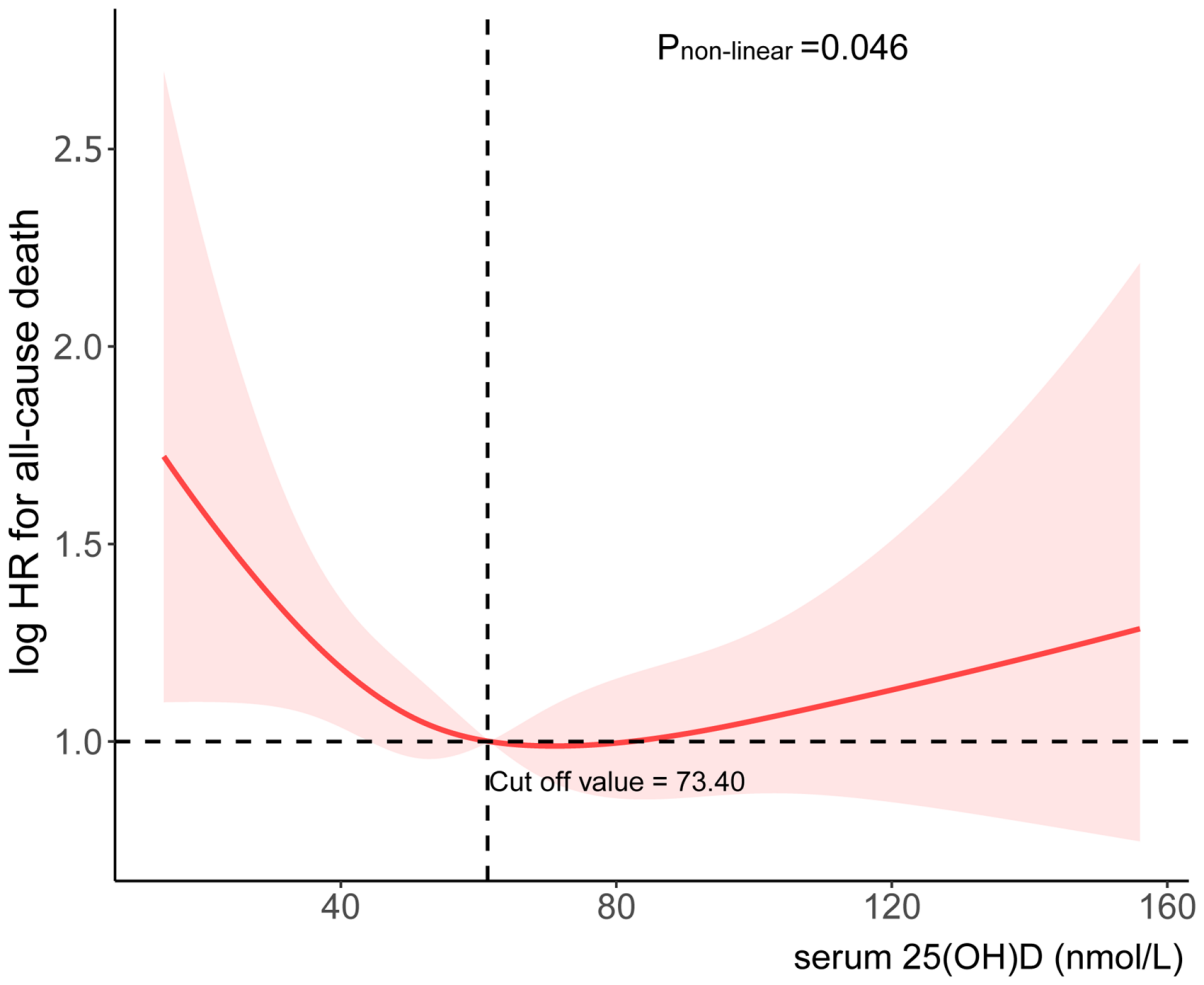
Association between serum 25(OH)D concentrations and risk of all-cause mortality.

We conducted additional research into the correlation between mortality risk in individuals suffering from chronic diarrhea and 25(OH)D levels. In Figure 3, there is a clear non-linear relationship between 25(OH)D levels and risk of death. The risk ratios showed a clear “L” shaped trend as the concentration changed. The threshold effect analysis showed that the relationship between 25(OH)D concentrations and mortality risk exhibited an inflection point at 73.40 nmol/L. A reduction of 2.2% in all-cause mortality was associated with every unit increase in 25(OH)D below 73.40 nmol/L (HR 0.98; 95% CI: 0.97, 0.99) (Table 3). There was no significant association with all-cause mortality at 25(OH)D concentrations above 73.40 nmol/L (HR 1.00; 95% CI: 0.99, 1.01). This “L” shaped relationship may indicate that excess 25(OH)D is not beneficial and that maintaining appropriate levels is essential to reduce the risk of death.

**Table 3:** Nonlinear association between serum 25(OH)D concentrations and all-cause mortality in patients with chronic diarrhea.

All-cause mortality	Adjusted HR (95% CI)	*P*-value
Inflection point (nmol/L)	73.40	–
Per 1-unit increment in 25(OH)D concentrations ≤73.40 nmol/L	0.98 (0.97, 0.99)	<0.01
Per 1-unit increment in 25(OH)D concentrations >73.40 nmol/L	1.00 (0.99, 1.01)	0.45
Difference in effect between ≤73.40 and > 73.40 nmol/L	1.03 (1.01, 1.04)	<0.01
Predicted value at the inflection point	−1.81 (−1.97, −1.64)	–
Log-likelihood ratio test	–	<0.01

### 3.4 Stratified analyses

In patients with chronic diarrhea, the effect of 25(OH)D concentrations on survival was consistent regardless of age, gender, race, body mass index, or tumor history, and there were no significant interactions between stratification variables. These findings indicate that, despite not reaching significance for interaction, the association between 25(OH)D levels and all-cause mortality is stronger in middle-aged adults (≥45, <60 years), females, non-Hispanic white people, normal-weight individuals (BMI <25), and patients with chronic diarrhea who had no history of cancer.

## 4 Discussion

In this study, we found an L-shaped relationship between 25(OH)D levels and all-cause mortality in patients with chronic diarrhea. A significant increase in all-cause mortality was observed when 25(OH)D concentrations were below 73.40 nmol/L, whereas mortality stabilized when concentrations were above this level. This finding suggests that lower 25(OH)D levels are significantly associated with increased all-cause mortality and that this association is independent of traditional risk factors such as stroke, diabetes, hypertension, cancer, cardiovascular disease, BMI and lifestyle. Even after adjusting for these factors, 25(OH)D still had a significant impact on all-cause mortality. The findings may be used to develop dietary guidelines and clinical interventions to reduce all-cause mortality in chronic diarrhea patients.

Our study indicates that 71.94% of patients with chronic diarrhea have insufficient 25(OH)D levels, highlighting a prevalent vitamin D deficiency in this population. In patients with chronic diarrhea, 25(OH)D concentrations and mortality all cause are associated in a significant L-shaped manner (Figure 3). When 25(OH)D concentrations are below 73.40 nmol/L, the death risk increases significantly, while it remains relatively stable above this level. According to these findings, 25(OH)D may be protective of health if it is below certain thresholds, which is consistent with other studies finding a non-linear relationship between 25(OH)D and mortality. Once the level of 25(OH)D reaches a certain threshold, mortality does not decrease further.

Vitamin D is well acknowledged for its crucial contribution to gut health. Its ability to modulate the immune system plays a significant role in maintaining the well-being of the gastrointestinal tract (21, 22). A systematic evaluation and meta-analysis showed that the prevalence of vitamin D deficiency was significantly higher in patients with IBD, with a 64% higher prevalence of vitamin D deficiency compared to controls. This association was particularly pronounced in individuals with ulcerative colitis (UC) (23). It has been shown that chronic diarrhea can be caused by inflammation of the bowels (24, 25). Immune and inflammatory processes are essential in the development of chronic diarrhea. Activation of inflammatory mediators is essential in the progression of chronic diarrhea (26–28).

A key factor in intestinal inflammation is the increased presence of immune cells, including choriocytes and mast cells, along with the release of 5-hydroxytryptamine, reactive oxygen species, cytokines, and the induction of oxidative stress (28, 29). Inflammation leads to degeneration and loss of intestinal neurons, which in turn impairs the structure and function of the enteric nervous system (ENS) (30–32), and this damage may trigger intestinal dysfunction leading to chronic diarrhea. ENS abnormalities affect intestinal motility and secretory function (30, 33), this leads to persistent diarrheal symptoms. The anti-inflammatory properties of vitamin D are thought to help restore the function of ENS (34, 35), thereby normalizing bowel function and controlling diarrheal symptoms. This can be primarily linked to the presence of vitamin D receptors within the nervous system, which are crucial for the production, upkeep, and regulation of neurotransmitters (14, 36).

Numerous studies have shown that increased 25(OH)D levels is correlated with a reduced risk of all-cause, cardiovascular, and cancer mortality (37–39). A cohort study showed that elevated 25(OH)D levels were linked to a lower risk of both all-cause mortality and cardiovascular disease (CVD) mortality among the general population in Korea. However, no significant association was identified between this level and cancer mortality (40). Differences in the results of different studies may stem from ethnic and lifestyle differences in the study populations, differences in definitions and measurement criteria for 25(OH)D levels, differences in follow-up time and study design, differences in the degree of control for confounders, the influence of cultural and environmental factors, and differences in statistical methods. These factors collectively influence the relationship between disease mortality and 25(OH)D. A meta-analysis examining 17 studies focused on colorectal cancer patients found a link between higher 25(OH)D levels and lower mortality rates. In particular, every 20 nmol/L rise in 25(OH)D levels was associated with a 7% decrease in the overall risk of death (41).

**Figure 4:**
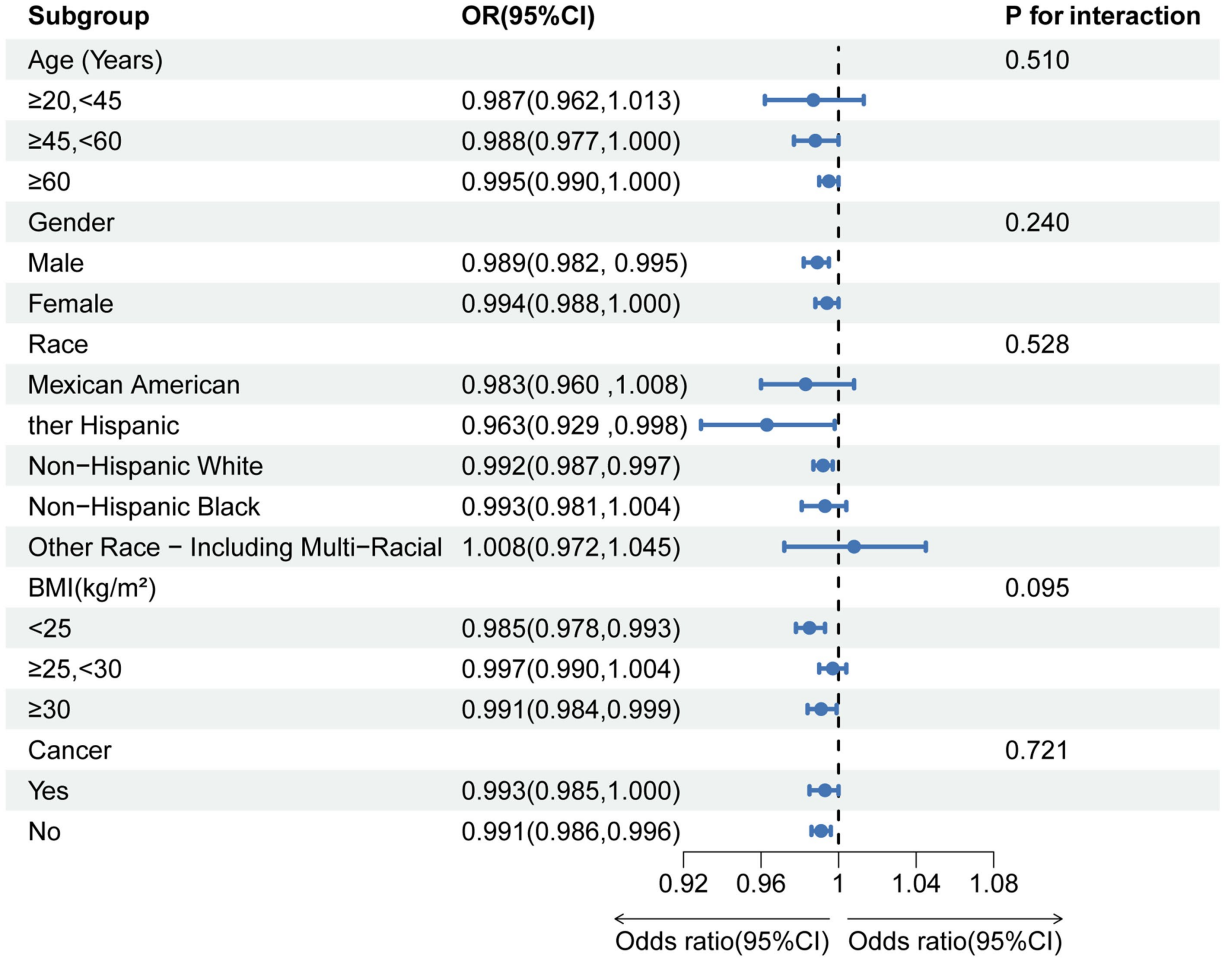
Subgroup analysis of the association between serum 25(OH)D concentrations and chronic diarrhea.

A forward-looking cohort study revealed a complex connection between 25(OH)D levels and the overall risk of mortality in individuals with inflammatory bowel disease. Notably, among female patients with 25(OH)D concentrations between 44 and 78 nmol/L, mortality rates were significantly reduced (22). A separate cohort study examined the connection between 25(OH)D levels and the risk of 16 different cancers, and cancer-related deaths and overall mortality in individuals with metabolic syndrome. The findings indicated that elevated 25(OH)D levels were linked to a reduced risk of developing colon, lung, and kidney cancers. Nevertheless, no notable connections were observed with other cancer types (42). These results indicate that vitamin D offers various health advantages for particular groups, potentially benefiting individuals suffering from chronic diarrhea as well, thereby reinforcing its protective function in certain long-term illnesses. Moreover, our results align with the discovery of a nonlinear “L-shaped” relationship between 25(OH)D levels and overall mortality (Figure 4).

To better pinpoint populations that are at a heightened risk of overall mortality among patients suffering from chronic diarrhea, we conducted subgroup analyses. The findings indicated that patients with elevated 25(OH)D levels (≥50.00 mmol/L), middle-aged adults (≥45, <60 years), white race, normal BMI (<25), and no history of cancer had a more pronounced survival advantage in terms of all-cause mortality. The non-linear relationship appears to primarily pertain to males in our research. However, a study examining adult patients suffering from inflammatory bowel disease revealed that female patients with elevated levels of 25(OH)D experienced reduced overall mortality rates (22). Whether our findings are incidental still requires further research to confirm.

## 5 Limitations

This research comes with a few notable limitations. To begin with, the definition of chronic diarrhea relies on self-reported data from participants, which may lead to information bias and hinder the accurate assessment of actual diarrhea conditions. Secondly, patients with self-reported inflammatory bowel disease, celiac disease, and colon cancer were excluded from the study, which may place limitations on the applicability of the findings to the broader population of patients with chronic diarrhea. Although we have adjusted for multiple known confounders such as BMI, age, and sex, there may still be unmeasured or unadjusted potential confounders, including dietary habits, environmental factors, and other health conditions. We also recognize that nutritional status may be another key factor influencing BSFS and mortality outcomes. Nutritional intake may not only affect gut function and immune responses, but may also exacerbate gastrointestinal symptoms and increase the risk of mortality. However, data limitations prevented us from fully accounting for nutritional status in our analyses. In addition, 25(OH)D levels in NHANES were only assessed at specific time points, which meant that we were unable to capture their long-term changes. The time delay in follow-up data also suggests that initial measurements may not fully reflect fluctuations in patients’ health status throughout the course of the study. As a cross-sectional study, we were unable to determine a causal relationship between 25(OH)D levels and all-cause mortality. Although the study found a significant association, this does not mean that low serum 25(OH)D levels are a direct cause of increased mortality. Limitations of the cross-sectional design prevented us from assessing the effects of long-term exposure and from completely excluding other potential confounders. Therefore, future prospective studies or randomized controlled trials will be more helpful in validating this relationship and further exploring the potential impact of 25(OH)D supplementation on mortality in patients with chronic diarrhea.

## 6 Conclusion

This research is the inaugural effort to investigate the connection between 25(OH)D levels and overall mortality in individuals suffering from chronic diarrhea. In the United States, individuals with chronic diarrhea exhibited a notable and non-linear relationship between reduced 25(OH)D levels and an increased likelihood of mortality from all causes, with 73.40 nmol/L potentially representing a critical threshold. While these findings provide valuable insight into the potential role of vitamin D in the management of chronic diarrhea, it is important to recognize that this study only demonstrates an association, not causation. Thus, while increased sun exposure and vitamin D supplementation may hold promise for patients with chronic diarrhea, further prospective studies are needed to confirm their impact on mortality.

## Data Availability

Publicly available datasets were analyzed in this study. This data can be found at: https://www.cdc.gov/nchs/nhanes/index.htm, National Health and Nutrition Examination Survey (NHANES).
